# Enhancing bulb yield through nitrogen fertilization and the use of hybrid onion (*Alluim cepa* L.) varieties in northwest Ethiopia

**DOI:** 10.1371/journal.pone.0312394

**Published:** 2024-10-24

**Authors:** Yebirzaf Yeshiwas, Melkamu Alemayehu, Enyew Adgo

**Affiliations:** Bahir Dar University College of Agriculture and Environmental Sciences, Bahir Dar, Ethiopia; United States Department of Agriculture, UNITED STATES OF AMERICA

## Abstract

Onions are among the most important cash crops in developing countries, including Ethiopia. However, its production and productivity are very low, which is associated with inappropriate fertilization and the use of low-yielding varieties. Therefore, the present study was conducted to evaluate the effects of the nitrogen fertilizer rate on the growth, yield, and quality of hybrid onion varieties in northwest Ethiopia. The experiment was conducted at three locations (Koga, Woreta, and Woramit) during the 2021/2022 cropping season under irrigated conditions. The treatments consisted of three hybrid onion varieties (Russet, Jambar, Red Coach) and one open-pollinated onion variety (Bombay Red) and four nitrogen rates (0, 41, 82, and 123 kg ha^-1^), which were laid out in a randomized complete block design with a factorial arrangement of 4*4 in three replications. The results of the present study revealed that onion growth, yield and quality were influenced by the nitrogen fertilizer rate and onion variety across all locations. Compared with the open pollinated Bombay Red variety, the hybrid varieties (Russet and Jambar) performed well in terms of bulb diameter, bulb weight, total yield, marketable bulb yield, and pungency. Nitrogen fertilizer applied at a rate of 82 kg ha^-1^ resulted in the highest growth and yield parameters of onion. The Russet and Jambar varieties recorded the highest marketable bulb yields of 26.50 t ha^-1^ and 24.57 t ha^-1^, respectively. Onion varieties treated with the highest nitrogen fertilizer dosage of 123 kg ha^-1^, particularly the Bombay Red variety, exhibited the longest duration to reach maturity. Onion plants supplied with 82 kg ha^-1^ nitrogen presented the highest marketable bulb yields, with a value of 26.77 t ha^-1^. Too much nitrogen above 82 kg ha^-1^ leads to decreased yield; hence, excess nitrogen is lost to the environment. Furthermore, the Jambar and Russet hybrid varieties and the application of 82 kg ha^-1^ nitrogen fertilizers provided the highest net benefit. The hybrid varieties Jambar and Russet and the application of 82 kg ha^-1^ nitrogen fertilizer can be recommended for onion production in the study area and areas with similar agroecosystems. Since this study is the first of its kind, considering other hybrid onion varieties and optimizing agronomic practices such as spacing and phosphorus fertilizer are also recommended in future research.

## 1 Introduction

Onion (*Alluim cepa L*.) is a vital vegetable crop worldwide [1]. It can be grown in different climatic zones and is widely cultivated for its edible bulbs and lower stem sections [2]. It was first introduced to the Ethiopian agricultural system in the 1970s by foreigners [3]. Onions are used to flavor and season a wide variety of dishes and are valued for their medicinal properties in many communities, including potential health benefits to minimize high blood pressure and reduce the risk of cancer, other heart diseases, and diabetes. Onions are rich in essential nutrients such as vitamin C, B6, biotin, chromium, calcium, and dietary fiber. When onions are sliced or crushed, they release an enzyme called alliinase, which leads to tearing or crying [4–6].

Onions are being grown in more than 130 countries around the world. The annual global production and productivity of onion are 99.97 million tonnes and 19.25 t ha^-1^, respectively [7]. China is the world’s top producer, followed by India and the US. In Africa, Egypt is the leading country, producing 22.08 million tons of onion per year for domestic and international markets, ranking fourth among world producers. Ethiopia ranks as the third largest onion producer on the African continent, trailing behind Egypt and South Africa [8]. Onion is mostly produced by small-scale farmers as a cash crop in Ethiopia. Despite the presence of a suitable environment and edaphic conditions, the production and productivity of onion in the country are very low. According to the CSA [9], the annual production and productivity of onion in Ethiopia are 346048.1 tons and 8.88 t ha^-1^, respectively. This is significantly lower than the potential yield of 40 t ha⁻^1^ achieved under research station conditions [9]. Farmers in Ethiopia use open-pollinated varieties with low yields (Bombay Red and Adama Red). They also practice improper agronomic practices, including inappropriate soil fertility management [10,11]. These similar malpractices contribute to the low level of onion productivity and bulb qualities, such as total soluble solids (brix) and pungency, which exist in Ethiopia, affecting market potential and consumer preference.

Successful onion production depends on several factors, including the selection of adaptable high yielder varieties suited to local conditions, the implementation of proper agronomic practices and the reduction of postharvest losses [11,12]. Compared with open-pollinated varieties, hybrid onion varieties generally boast greater yield potential, offering uniform bulb sizes and enhanced storability, thereby extending shelf-life [13–15]. In the context of improving onion production in northwest Amhara Ethiopia, various hybrid varieties such as Russet, Red Coach, and Jambar have been introduced into the local farming system. These varieties were selected due to their high yield potential and better storability compared to open pollinated varieties. Preliminary trials and research conducted in similar agro-climatic regions have shown that these varieties adapted and perform well under local conditions of northwest Amhara Ethiopia [11]. However, despite their introduction, the full production technologies, particularly regarding their specific nitrogen fertilizer requirements, has not been comprehensively studied and require interventions.

Fertilization should aim to improve onion productivity and quality with respect to nutrient requirements related to optimal yield at a minimum cost and high efficiency of fertilization use [16]. Compared with most other crops, onion plants have shallow and unbranched roots, which increase their vulnerability to nutritional deficiencies [17]. Consequently, onions require a relatively high level of nitrogen and respond well to supplemental nitrogen fertilizer [17,18]. The rate of fertilizer application depends on various factors, such as the crop type, environmental conditions, soil fertility status, method of fertilization, and yield potential of the crop [19,20]. Dinega et al. [21] reported that intensifying nitrogen fertilizer management can improve yield and bulb quality in onion.

Although exogenous nitrogen application is known to increase onion yield, many researchers have reported that the application of high levels of nitrogen fertilizer leads to a reduction in the storage life of onion bulbs [22]. Excessively high doses of nitrogen also cause a delay in bulb maturity and encourage bolting, which is an undesirable characteristic [23]. Moreover, according to Khan et al. [24], a low rate of nitrogen causes a low yield of onions due to a shortage of nitrogen required for the chlorophyll pigment that is responsible for photosynthesis. Excessive nitrogen is hazardous to the environment, weakens foliage and predisposes plants to pathogenic diseases [25]. The application of the optimal nitrogen fertilizer rate is therefore essential in onion production [21,26]. In this context, this study aimed to optimize nitrogen fertilizer rates for the economical production of hybrid onion varieties in northwestern Ethiopia.

## 2. Materials and methods

### 2.1. Description of the study area

**Fig 1:**
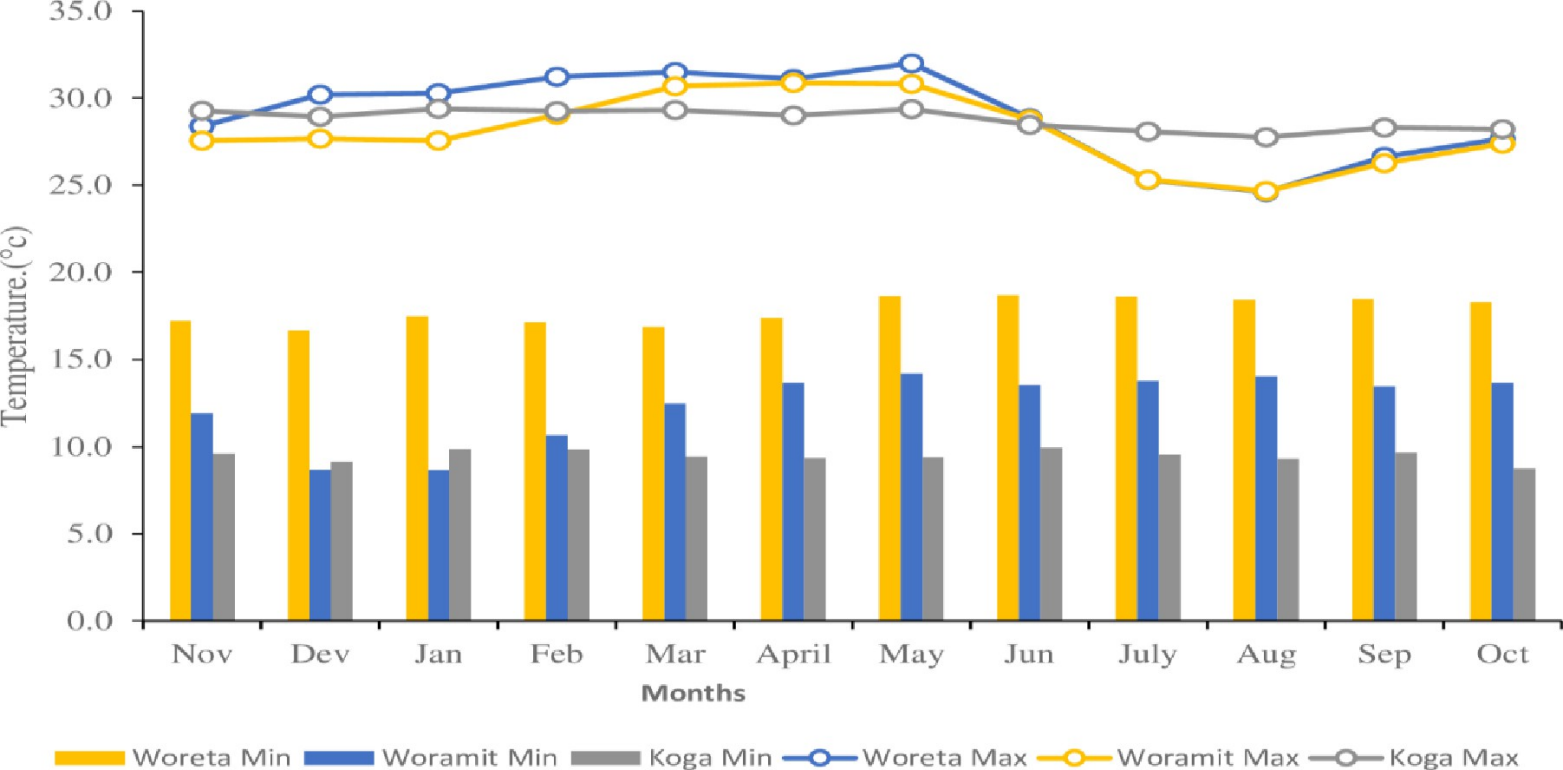
Minimum (lower) and maximum (upper) temperatures of the testing sites during the cropping season of 2021/2022. Source: Regional Meteorological Station [27].

Field experiment was carried out at three locations, namely, the North Mecha (Koga), Fogera (Woreta) and Bahir Dar Zuria (Woramit) districts of the Amhara region, during the 2021–2022 irrigation season. The geographical locations, altitudes, average, minimum and maximum temperatures, rainfall, and soil types of the districts are presented in Table 1. The average maximum and minimum temperatures of Woreta and Woramit during the experimental period were 29°C and 17.8°C and 28°C and 12.4°C, respectively. The Koga experimental site had the mean maximum and minimum temperature of 28.8°C and 9.5°C, respectively, as indicated in Fig 1 [27].

**Table 1:** Geographical locations and environmental conditions of the experimental sites (long years average).

Location	Lat. (N)	Long.(E)	Altitude (m.a.s.l)	Average (°C)		Rainfall (mm)	Soil texture
				Min.	Max		
Woramit	11°38′	37°10′	1800	13	25.5	1248.8	Nitisole
Koga	11°20ʹ	37°7ʹ	1953	9.7	26.8	1300–1380	Nitisole
Woreta	11°55′	37°42′	1828	12	28	1250	Vertisoil

#### Selected properties of the soil in the experimental locations

Soil samples from the experimental sites were collected via standard procedures prior to planting at each site. A composite soil sample was taken before the experimental plot was plowed by mixing samples collected at eight different locations along the two diagonal lines of the field at 0–20 cm depth via an auger. The physical and chemical properties of the composite soils from each location were analyzed at the Soil and Plant Nutrition Laboratory of the College of Agricultural and Environmental Sciences, Bahir Dar University. The results of the soil analysis are presented in Table 2.

**Table 2:** Status of nutrients in the experimental locations before planting in 2021/2022 irrigation season.

Soil properties	Soil Sample		
	Koga	Woramit	Woreta
**Texture**			
**Sand (%)**	56	44	43
**Silt (%)**	16	24	15
**Clay (%)**	28	32	42
**Textural class**	Sandy Clay Loam	Clay Loam	Clay
pH_H_2_O (1:2.5)	5.40	5.69	6.02
EC **(μS/cm)**	161.30	89.10	74.00
Organic Carbon **(%)**	2.18	1.72	1.33
Organic Matter (**%)**	3.77	2.96	2.29
CEC **(cmol (+) kg**^**-1**^**)**	27.60	26.00	41.40
Total Nitrogen **(%)**	0.42	0.17	0.18
Available Phosphorus (mg/kg) [Olsen method]	10.02	20.24	6.48

where: EC = electrical conductivity; CEC = cation exchange capacity.

The textural class of the experimental soil was found to be sandy clay loam in Koga, clay loam in Woramit and clay in Woreta, with pH values of 5.4, 5.69 and 6.06, respectively. The pH of Woreta falls within the optimal range, whereas those of Koga and Woramit are slightly acidic and are within a preferred range for most crops. The electrical conductivity values of the experimental soils are within acceptable limits for onions, indicating that the sites had low salinity levels. The organic matter content of the site is in the medium range (2–4%) for Ethiopian soils. The total nitrogen content of the soils was found to be high in Koga and low in Woreta and Woramit. The Olsen extractable available phosphorus (P) content of the soil was 10.02 mg kg⁻^1^ in Koga (rated as medium), 20.24 mg kg⁻^1^ in Woramit (rated as medium), and 6.48 mg kg⁻^1^ in Woreta (rated as low). The cation exchange capacity of the soil was 27, 26, and 41, ranging from medium to high (Table 2).

### 2.2. Experimental materials

Three hybrid F_1_ onion varieties, Russet, Red Coach, and Jambar, and one open pollinated Bombay Red variety as local check, were used as treatments in the present study. The seeds of the hybrid varieties were collected from commercial suppliers (Eshet Agricultural Input Supplier), while the Bombay Red variety seeds were obtained from the Melkassa Agricultural Research Center (MARC). The onion varieties were selected on the basis of the results of the preliminary pilot survey in which the farmers preferred them on the basis of their growth and yield performance. The general characteristics of the selected onion varieties are presented in Table 3.

**Table 3:** List of varieties and their descriptions.

Variety name	Year of release/registration	Area of adaptation		Maturity days
		Altitude (m)	Rainfall (mm)	
Russet F_I_	2013	1500–2200	Irrigated	90–110
Red Coach F_I_	2015	500–2200	Irrigated	107–120
Jambar F_I_	2011	1000–2500	Irrigated	90–110
Bombay Red	1980	700–2000	Irrigated	110–120

Source: MoARD [28–30].

### 2.3. Treatments and experimental design

The treatments consisted of four onion varieties (Russet, Red Coach, Jambar and Bombay Red), four nitrogen fertilizer rates (0 kg ha^-1^, 41 kg ha^-1^, 82 kg ha^-1^, and 123 kg ha^-1^), and three locations. The treatments were laid out in a randomized complete block design (RCBD) in a 4*4 factorial arrangement with three replications. Each treatment combination was randomly assigned to the experimental units within a block at each location. In total, 16 treatments were used in the experiment. The total plot size of the experiment was 1.6 m × 1.6 m (2.56 m^2^), while the net plot size was 1.8 m^2^. The blocks were separated by one meter, and the space between each plot within a block was 0.5 m.

### 2.4. Experimental procedures

Seeds of each variety were sown on well-prepared raised beds and mulched with straw. After germination, the mulching material was removed. Watering was done using a fine watering can where the frequency was determined on the basis of climatic conditions. The seedlings were maintained for 55 days after sowing and hardened before being transplanted to the field. This was done by reducing the frequency of watering and making the soil have a low moisture status when the seedlings were ready for field planting.

Healthy and vigorous 12–15 cm tall seedlings were carefully raised for transplanting. The selected seedlings were transplanted in January 2022 with a recommended double row spacing of 40 cm × 20 cm × 10 cm [28].

All agronomic practices were applied according to the recommendation made for onion [28]. The nitrogen fertilizer based on the rates of the treatments was applied in the form of urea (46% N) in two equal splits, where the first 50% of the nitrogen was applied 15 days after transplanting, while the remaining 50% was applied one month after transplanting. Triple superphosphate (46% P2O5) was used as a source of phosphorus and was applied at a rate of 92 kg ha^-1^ P_2_O_5_ at the time of uniform transplantation for all the plots [31].

Irrigation water was applied at four-day intervals via furrow irrigation for the first four weeks and then extended to seven-day intervals until the physiological maturity stage. The application of irrigation water was completely stopped 15 days before harvesting for curing purposes. Onion bulbs were harvested when 70% of the plants in each plot showed neck fall [28,32].

### 2.5. Data collection

Measurements of onion growth, yield, and quality parameters were recorded from the central four rows of the net plot area at the physiological maturity stage and harvesting time. Plant height, leaf number per plant and leaf diameter were measured from ten randomly selected plants from the central four rows in each plot, whereas days to physiological maturity, bulb diameter, average bulb weight, total, marketable, and unmarketable bulb yields were collected in the net plot area.

**Plant height (cm)** was measured using ruler from the soil surface to the tip of the longest mature leaves at physiological maturity. **Leaf number per plant:** the total number of leaves per plant at physiological maturity. **Leaf diameter (cm):** refers to the maximum diameter of the longest leaf measured with a caliper at the widest point of the leaf. **Days to physiological maturity** refers to the actual number of days from transplanting to a day at which more than 70% of the plants in a plot showed yellowing of leaves. **Bulb diameter (cm)** was measured at harvest via a digital Vernier caliper at the widest point in the middle portion of the mature bulb. **The average bulb weight (g)** was computed by weighing ten marketable bulbs together and recording the average value in grams per plant using in [Eq 1] [32].


ABW=Totalbulbweight/Totalbulbnumber [1]

**The total bulb yield (ton ha**^**-1**^**)** was the sum of total marketable and unmarketable bulbs and was computed on the basis of the weight of mature bulb yield per net plot area and expressed in tons per hectare basis. **The marketable bulb yield (ton ha**^**-1**^**)** was determined after discarding bulbs smaller than 20 g, which were split, damaged, rotted and discolored. **The unmarketable bulb yield (ton ha**^**-1**^**)** was determined by categorizing the bulb as follows: undersized (<20 g), diseased, decayed, and physiologically disordered, such as split, were weighed and expressed as unmarketable bulbs. **Total soluble solid (TSS) (**°Brix**): TSS content** was determined via the procedures described by Waskar *et al*. [33]. The juice of the sample bulbs was extracted via a juice extractor, and the extracted juices were filtered using sieve. The TSS was determined using ATAGO® PR-32α with a range of 0 to 32% by placing one to two drops of clean juice on the prism at a monthly interval. The refractometer was standardized with distilled water (0% TSS). The prism of the refractometer was washed with distilled water and dried before the next use. **Firmness (Newton):** was determined with a portable fruit penetrometer (GY-4 model S/N G2001707622) using an 8 mm stainless steel punch. The bulb was penetrated at two opposing points on its equatorial region, then the average of the two measurements was taken. It was measured as the force in Newtons required to penetrate the scale. **Pungency (D. mol/ml)**—content of pyruvic acid developing in homogenized bulb tissue was used as measure of pungency following the modified procedure of Anthon and Barrett [34].

### 2.6. Data analysis

The collected data were first checked for meeting with all ANOVA assumptions (normality and homogeneity) after the Leven homogeneity test. Data were subjected to analysis of variance (ANOVA) using the PROC GLM model of SAS computer software version 9.0 (SAS Institute Inc., 2008).

Following Leven’s homogeneity test, a combined analysis of variance was performed using the SAS PROC MIXED procedure. The nitrogen fertilizer rate and variety were considered as fixed effects, whereas locations were considered as a random effect. Whenever ANOVA was significant (*P <0*.*05)*, mean separation was carried out using least significant difference (LSD) tests [35]. Multiple regression analysis was conducted to determine nitrogen coefficients to develop the model using the PROC REG procedure for marketable bulb yield. The quadratic fit model was used to estimate the optimum values of nitrogen above which no additional net returns were predicted.

### 2.7. Partial budget analysis

Partial budget analysis following the procedures described by CIMMYT [36] was performed to evaluate the economics of the treatments. The marketable yield was downscaled by 10% since the experimental yields are usually higher than those of farmers. The gross benefits of the treatments were estimated by multiplying the adjusted marketable bulb yield with the farm-gate price (20.00 Birr kg^-1^) of onion bulb prevailing in the study area during harvest. The price of urea fertilizer in local marketplaces was 32.50 ETB kg^-1^. Furthermore, the prices of onion seeds (Russet and Jambar 16,000 ETB kg^-1^, Red Coach 13,000 kg^-1^, and Bombay Red 2,500 ETB kg^-1^) and labor cost (150.00 ETB day^-1^) for fertilizer application were used to calculate gross incomes and variable costs. Seed cost, labor cost and urea fertilizer cost are considered variable costs that change with the treatment. The sum of these costs was considered as the total variable cost of the treatment. Net benefits from treatments were obtained by deducting total variable costs from gross benefit on a hectare basis, as indicated by CIMMYT [36].

## 3. Results and discussion

### 3.1. Effects of the nitrogen fertilizer rate on the phenology and vegetative traits of onion varieties

The main effects of variety and nitrogen rate significantly *(P< 0*.*01)* influenced days to physiological maturity, plant height, and leaf number plant^-1^. However, the two-way interactions between variety and nitrogen did not influence (*p>0*.*05*) the number of days needed for the physiological maturity of the onion (S1 Table).

The Russet onion variety matured earlier (94.7 days), whereas Bombay Red took the longest number of days (108.47) to achieve physiological maturity across the three locations (Table 4). The variation in the days required to reach maturity between onion varieties could be due to their genetic differences. Different scholars have reported similar results, where varieties differ in the number of days required to reach physiological maturity [37–39]. The application of fertilizer also significantly extended the physiological maturity of onion across locations.

**Table 4:** Effects of variety, nitrogen fertilizer on maturity and growth of the onion across the locations.

Variety	Days to physiological maturity (Days)	Plant height (cm)	Leaf number plant^-1^
Russet	94.69^c^	48.42b	9.02bc
Jambar	100.97^b^	50.17a	9c
Red Coach	101.72^b^	51.12a	9.44ab
Bombay Red	108.47^a^	51.42a	9.69a
*P values*	**	**	**
LSD _0.05_	3.27	1.70	0.41
**Nitrogen (kg ha** ^**-1**^ **)**			
0	100.13^b^	46.48c	8.63c
41	100.55^b^	50.23b	9.09b
82	100.94^b^	52.03a	9.61a
123	104.22^a^	51.42a	9.82a
*P values*	**	**	**
LSD _0.05_	2.7	3.66	0.41
CV (%)	5.6	15.61	11.95

Where

*** = p<0.001

** = P<0.01; and Ns = P>0.05. Means followed by the same letter within the same column are not significantly different at the 5% probability level; LSD_0.05_ = least significant difference at the 5% probability level; and CV = coefficient of variation.

Compared with those in the other treatments, onion plants supplied with 123 kg ha^-1^ nitrogen required the longest days (104.22 days) to reach physiological maturity, whereas onion plants without nitrogen fertilizer matured earlier (100.13 days) (Table 4). This could be attributed to the effects of nitrogen, which extends the vegetative growth period, which in turn delays the physiological maturity of onion plants. Nitrogen fertilizer also increases vegetative growth, which increases the interception of solar radiation and consequently prolongs the number of days needed to reach physiological maturity [40]. The results of the present study are in agreement with the findings of different scholars who reported excessive vegetative growth and delayed onion maturity with the application of relatively high levels of nitrogen fertilizer [17,41,42]. Similarly, Kebede [43] reported that shallot plants were influenced by nutrients and that increased nitrogen fertilizer rates enhanced vegetative growth, delayed maturity, and reduced bulblet size.

The results of the main effects indicated that the Jambar variety, Bombay Red, and Red Coach presented the highest and statistically similar plant heights, with measurement values of 51.12cm, 51.42cm, and 50.17cm, respectively. However, the Russet variety (48.14cm) recorded the lowest plant height (Table 4). The differences in plant height among the varieties could be attributed to genotypic differences, which is in line with previous findings [38,44–47]. The authors reported significant differences in plant height between onion cultivars because of their genetic variations. Compared with the hybrid Russet variety, the open-pollinated Bombay Red variety presented the longest plant height. This could be attributed to the specific breeding goals in which hybrids are prioritized for improved bulb yield rather than excessive vegetative growth compared with certain open-pollinated varieties [48,49].

The onion plants supplied with 123 kg ha^-1^ nitrogen recorded the longest plant height, which was statistically similar to the heights recorded with the application of 82 kg ha^-1^ nitrogen. The lowest mean plant height (46.48cm) was recorded for the plants grown without nitrogen fertilizer (Table 4). The application of 82 kg ha^-1^ nitrogen increased the plant height by approximately 11.9% compared with that of the plants grown without nitrogen. However, a further increase in the nitrogen rate did not significantly affect the height of the plant. The application of nitrogen plays a crucial role in cell division, elongation, and improvement of vegetative growth through increased photosynthetic areas of plants, including onions. The improvement in onion plant height through the application of nitrogen fertilizer has also been reported by different scholars [50–56].

The analysis revealed that the highest average leaf number per plant was recorded for the Bombay Red and Jambar varieties, with values of 9.69 and 9.44, respectively, which were statistically similar to each other. On the other hand, the lowest number of leaves per plant (9.0) was recorded from the Red Coach variety at the three locations, as indicated in Table 4. The difference in the number of leaves per plant could be due to the difference in the inherent genetic makeup of the onion varieties. The present findings align with previous results that reported differences in genetic makeup as the cause of variations in leaf number between onion varieties [57–59].

The application of 123 kg ha^-1^ and 82 kg ha^-1^ nitrogen resulted in the highest number of leaves per plant (9.82 and 9.61, respectively), whereas plants without nitrogen fertilizer produced the lowest number of leaves per plant (8.63), as indicated in Table 4. Nitrogen is an important nutrient necessary for the synthesis of different protein components that are required for leaf development. In this context, studies by Kokobe et al. [52] have revealed the positive impact of nitrogen on promoting the production of new shoots during the vegetative growth of plants, which may help increase onion leaf number. The application of nitrogen fertilizer also increased the leaf number of onion plants in different studies [42,44,60–62].

### 3.2. Influences of variety and nitrogen fertilizer on bulb yield and onion quality

The results of the combined analysis indicated that the bulb diameter, average bulb weight, total bulb yield, marketable bulb yield and unmarketable bulb yield were influenced by the main effects of variety and nitrogen rate. However, the interaction effect of variety and nitrogen rate was not significant (S1 Table).

The widest diameter of the onion bulb was recorded from the Russet (69.54 cm) and Jambar (68.53 cm) varieties, whereas the lowest diameter of the bulb (64.56 cm) was recorded from the Bombay Red variety (Table 5).

**Table 5:** Influence of variety and nitrogen rate on onion bulb size at three locations.

Onion varieties	Bulb diameter (cm)	Average bulb weight (g)
Russet	69.54a	93.12a
Red Coach	65.85b	91.16a
Jambar	68.53ab	94.28a
Bombay Red	64.56c	81.7b
*P values*	**	**
LSD_0.05_	3.82	3.68
**Nitrogen (kg ha** ^**-1**^ **)**	
0	60.44b	79.84d
41	63.99b	85.87c
82	73.4a	101.18a
123	70.66a	93.38b
*P values*	***	**
LSD_0.05_	3.82	3.19
CV (%)	12.22	8.74

Where

*** = p<0.001

** = P<0.01; and Ns = P>0.05. Means followed by the same letter within the same column are not significantly different at the 5% probability level: LSD_0.05_ = Least significant difference at 5%; and CV = coefficient of variation.

The widest diameter of the onion bulb was recorded from plots that received 82 kg ha^-1^ (73.4 cm) and 123 kg ha^-1^ (70.66 cm) onion bulb. On the other hand, the lowest bulb width of 60.44 cm was recorded from the control plot without nitrogen fertilizer. Compared with the control treatment without nitrogen, the application of nitrogen at a rate of 82 kg ha^-1^ increased the mean bulb diameter by 21.5% (Table 5). However, a further increase in the nitrogen rate above 82 kg ha^-1^ did not affect the mean bulb diameter of the onion. The diameter of the bulb is one of the most important yield components that indicates the yield potential of onion varieties. The parameter is influenced by the genetic makeup of the variety. The greater bulb diameter recorded from the hybrid varieties than from the open pollinated Bombay Red variety in the present study could therefore be attributed to the differences in the genetic makeup of the varieties [63,64]. Similar findings were also reported by Mekdes [65], where the open-pollinated Bombay Red variety presented the lowest bulb diameter. The increase in bulb diameter due to the application of nitrogen fertilizer is clearly associated with the growth-promoting effects of nitrogen, which improve dry matter production in onion plants. An increase in bulb size through the application of nitrogen has also been reported by different researchers [42,60,66–70].

The hybrid varieties Jambar (94.28 g), Russet (93.12 g) and Red Coach (91.16 g) produced the heaviest bulbs, whereas Bombay Red (81.7 g), an open pollinated variety, as indicated in Table 5.

Similarly, the application of 82 kg ha^–1^ nitrogen resulted in the highest bulb weight of onion, whereas the lowest bulb weight was recorded for plants without nitrogen fertilizer. The bulb weight is an important parameter that indicates the yield potential of a given onion variety. In this sense, the hybrid varieties in the present study produced relatively larger bulbs than did the open pollinated variety, which may be associated with their genetic makeup. The results of the present study are consistent with the findings of other researchers who reported that hybrid varieties produced relatively larger bulbs and thus higher bulb yields than did open pollinated onion varieties [49]. Compared with the control without nitrogen, nitrogen supplied at a rate of 82 kg ha^-1^ increased the bulb weight by 27%. However, additional applications of nitrogen beyond 82 kg ha^-1^ did not result in any significant changes in bulb weight.

The Russet (26.93 t ha^-1^) and Jambar (25.08 t ha^-1^) varieties presented the highest total bulb yields, whereas the Red Coach (25.08 t ha^-1^) and Bombay Red (20.45 t ha^-1^) varieties presented the lowest total bulb yields across the three locations.

The application of 82 kg ha^-1^ nitrogen recorded the highest total bulb yield (26.26 t ha^-1^), whereas plants without nitrogen fertilizer recorded the lowest total bulb yield (24.97 t ha^-1^), as indicated in Table 6. High doses of nitrogen result in excessive vegetative growth, which can cause self-shading and a reduction in photosynthesis activities, which in turn reduces the development of storage organs [71,72]. In contrast, a low level of nitrogen causes a reduction in photosynthetic activity associated with a reduction in chlorophyll content [73]. In this context, the application of nitrogen at an appropriate rate is necessary to enhance the leaf area and chlorophyll content and thus increase photosynthetic activity and yield, as indicated in the present study. Compared with the control treatment without nitrogen fertilizer, the application of 82 kg ha^-1^ nitrogen in the present study increased the total onion bulb yield by 38.5%. However, a further increase in the nitrogen rate did not significantly affect the total bulb yield. The yield potential of onion varieties depends on the genetic potential, environmental conditions, and management activities implemented [74,75]. In this sense, the differences in total bulb yield observed in the present study could be attributed to their genetic potential. The highest total bulb yield observed for the Russet and Jambar varieties could be associated with the relatively larger bulbs of the varieties observed in the present study. These findings indicate that these varieties have high nutrient utilization capacity, better adaptability, and high yield performance than do the other onion varieties. These results are in agreement with the findings of other scholars who reported that total bulb yield differences between onion varieties could be attributed to variations in nitrogen fertilizer response and genetic makeup, as well as in the interaction of genotypes and environmental effects [64,76,77]. According to Simon *et al*. [56], proper nutrient management positively contributes to onion production and productivity improvement, especially in areas where nutrient deficiency is critical.

**Table 6:** Effects of variety and nitrogen on onion bulb yield.

Onion varieties	Marketable bulb yield (t ha^-1^)	Unmarketable bulb yield (t ha^-1^)	Total dry bulb yield (t ha^-1^)
Russet	26.50a	0.43c	26.93a
Red Coach	21.84b	0.50b	22.35b
Jambar	24.57a	0.51b	25.09a
Bombay Red	19.86b	0.59a	20.453b
*P values*	***	***	***
LSD_0.05_	2.25	0.43	2.24
**Nitrogen rate (kg ha** ^**-1**^ **)**			
0	19.09c	0.58a	19.68c
41	22.39b	0.51b	22.91b
82	26.77a	0.48b	27.26a
123	24.51b	0.46b	24.97b
*P values*	***	***	***
LSD_0.05_	2.25	0.05	2.24
CV (%)	20.76	21.43	20.22

Where

*** = p<0.001

** = P<0.01; and Ns = P>0.05. Means followed by the same letter within the same column are not significantly different at the 5% probability level; LSD_0.05_ = least significant difference at the 5% probability level; and CV = coefficient of variation.

The highest marketable bulb yield was obtained from the Russet (26.50 t ha^-1^) and Jambar varieties (24.57 t ha^-1^). However, the lowest marketable bulb yield was recorded for the Bombay Red variety (19.86 t ha^-1^), which was statistically similar to that of the Red Coach variety.

**Fig 2:**
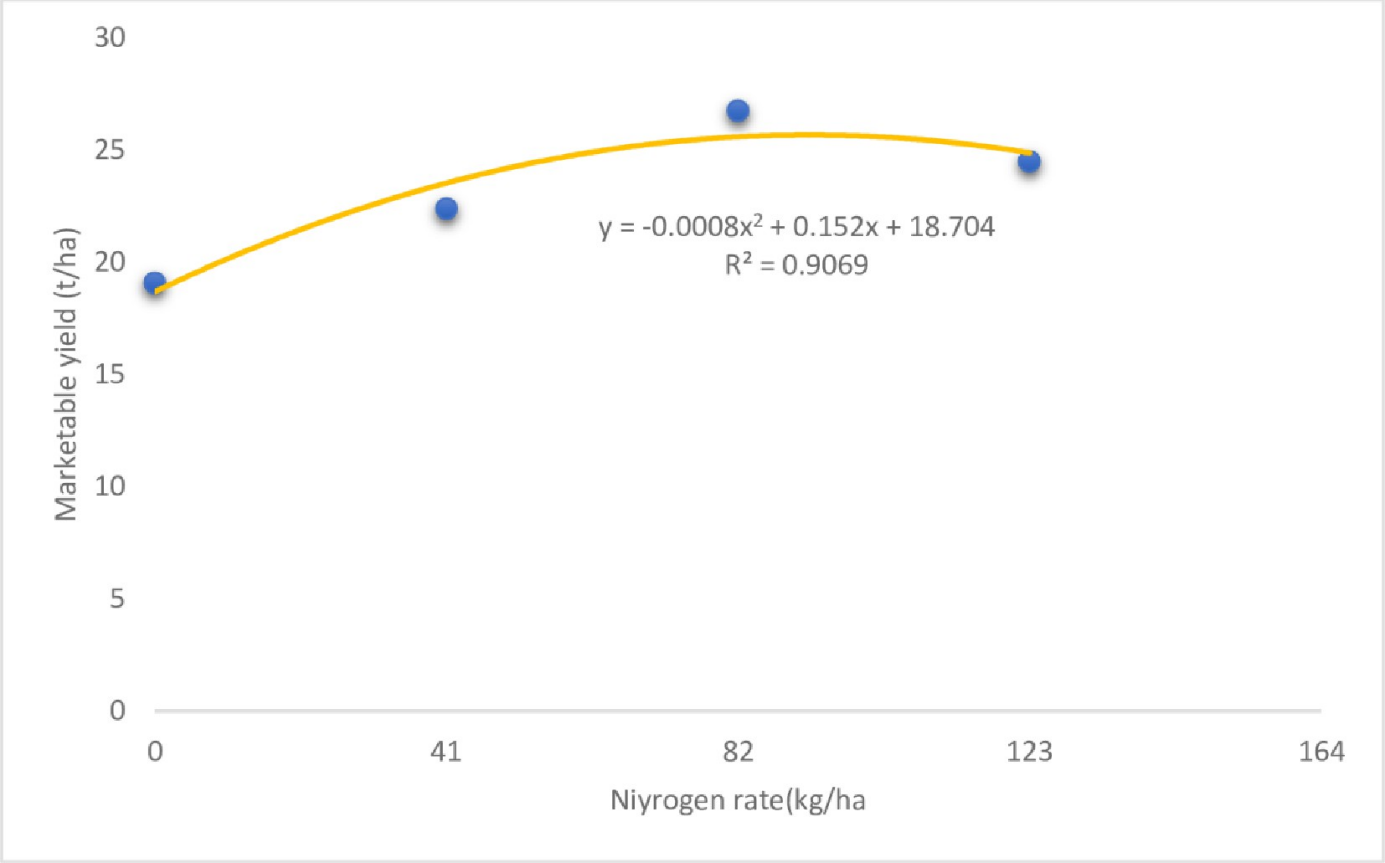
Effect of nitrogen rate on the marketable bulb yield of onion varieties.

The onion plants supplied with 82 kg ha^-1^ nitrogen produced the highest marketable yield (26.77 t ha^-1^), whereas the plants without nitrogen fertilizer produced the lowest marketable bulb yield (19.09 t ha^-1^) (Table 6). The increased marketable yield of hybrid onion varieties (Russet and Jambar) observed in the present study is clearly due to their genetic potential, as indicated by different scholars who reported marketable bulb yield differences between onion varieties [38,78–80]. Jilani *et al*. [81] and Yemane *et al*. [76] also reported that different onion varieties of the same species produced different yields because of their genetic makeup. Nitrogen fertilizer obviously influences crop yield, including onion yield. The marketable bulb yield of onion varies with the nitrogen fertilizer rate [82]. As illustrated in Fig 2, the marketable yield of onion in the present study generally tended to increase and reached a plateau at the 82 kg ha^-1^ nitrogen rate, which was found to be optimal for onion production at the study sites. However, a further increase in the nitrogen rate beyond 82 kg ha^-1^ did not significantly influence the marketable bulb yield; rather, it resulted in a decreasing trend in the marketable yield.

**Fig 3:**
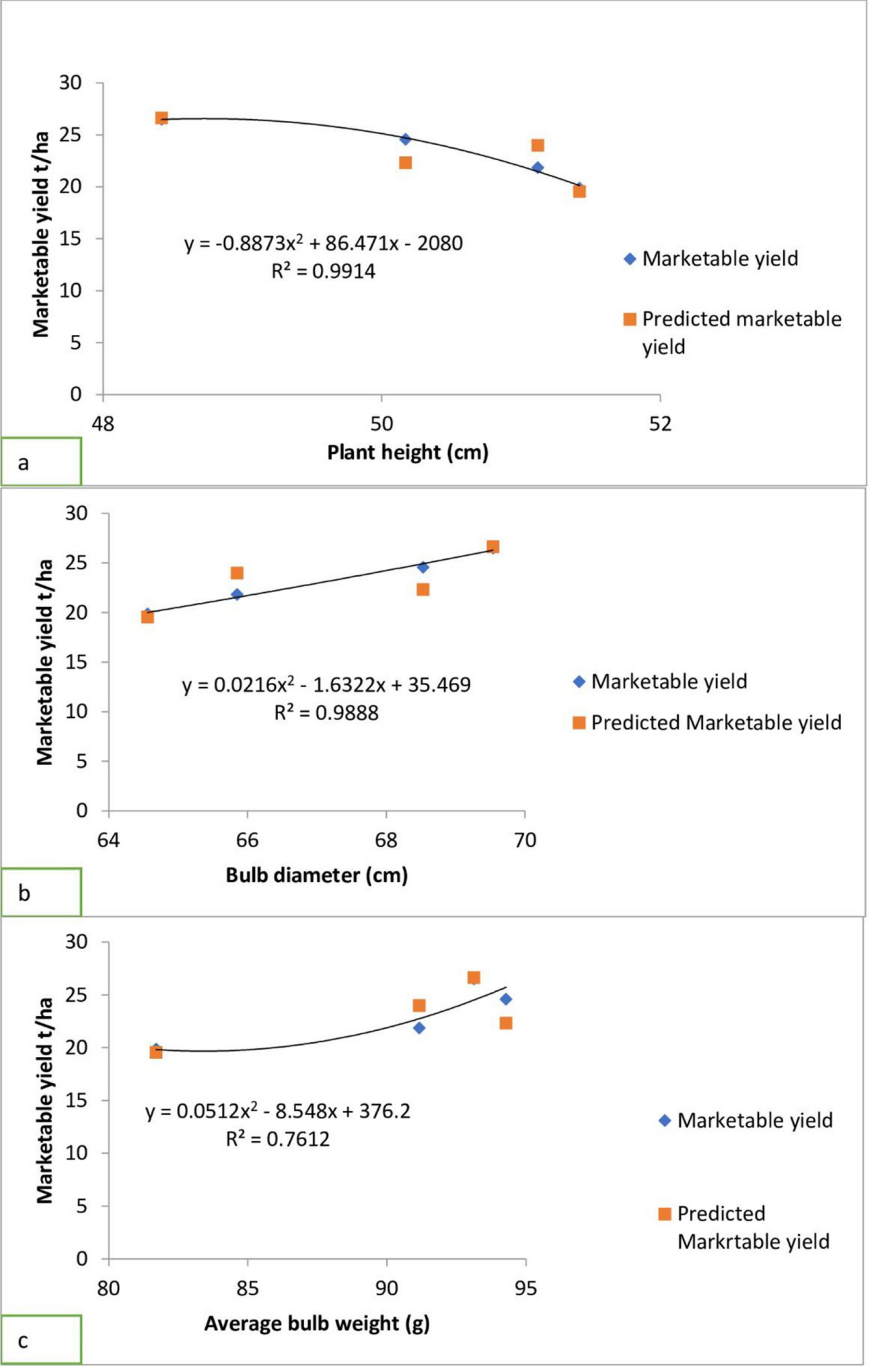
Relationship between marketable yield and traits (a) plant height, (b) bulb diameter (c) average bulb weight.

There was a decreasing trend and negative relationship between marketable bulb yield and plant height (Fig 3A), while an increasing trend and positive slope between marketable bulb yield bulb diameter (Fig 3B), and average bulb weight (Fig 3C).

The Bombay Red onion variety presented the highest unmarketable bulb yield per hectare (0.59 t ha^-1^), whereas the hybrid onion varieties generally produced the lowest unmarketable bulb yield per hectare. Compared with the plots supplied with nitrogen fertilizer, the unfertilized plot also presented the highest unmarketable bulb yield (Table 6), which could be attributed to the application of a suboptimal nitrogen supply that produced poor-quality bulbs that contributed to a higher unmarketable onion yield. This result agrees with the findings of Zeleke *et al*. [47], who reported the highest unmarketable bulb yield from unfertilized plots.

### 3.3. Influence of nitrogen fertilizer on the bulb quality of onion varieties

The analysis of variance revealed that the main effects, as well as the two-way interaction effects of variety and nitrogen, had highly significant (*p<0*.*01*) effects on the solid soluble content of onion bulbs. Onion pungency was influenced (*p<0*.*001*) by the main effects of variety and nitrogen rate. However, the two-way interaction of nitrogen*variety did not significantly *(p>0*.*05)* influence (S1 Table).

In terms of the interaction effect, the Bombay Red variety received 82 kg ha^-1^ nitrogen and 123 kg ha^-1^ nitrogen, resulting in the highest TSS levels of 12.36°Brix and 12.16°Brix, respectively (Table 7). Increasing the nitrogen rate improves plant growth and development through increased photosynthetic activities and dry matter production [83], which could also be the case in the present study. In this context, Al-Fraihat [51] reported that increasing the nitrogen fertilizer rate from 100 kg N ha^-1^ to 200 kg N ha^-1^ increased the TSS from 13.75% to 14.70%. Morsy *et al*. [55] also reported that the application of 120 kg ha^-1^ N led to the highest values of TSS, whereas the application of 90 kg nitrogen ha^-1^ resulted in the lowest value of TSS. On the other hand, the differences in the TSS values observed in the present study could be attributed to the genetic makeup of onion varieties, as indicated by Chope *et al*. [84]. Generally, high-yield varieties with larger bulb sizes (higher bulb weights) have lower TSS contents than varieties with small bulbs and lower yields [85], which is the case for the Bombay Red variety in the present study.

**Table 7:** TSS of onion varieties influenced by the interaction of variety and nitrogen.

Varieties	Nitrogen rate (kg ha ^-1^)	TSS (°Brix)
Russet	0	6.86^g^
	41	7.98^ef^
	82	7.44^fg^
	123	7.70^fg^
Red Coach	0	7.66^fg^
	41	7.90^efg^
	82	8.10^ef^
	123	8.93^de^
Jambar	0	8.16^ef^
	41	9.63^d^
	82	9.30^d^
	123	9.90^cd^
Bombay Red	0	10.76^bc^
	41	11.07^b^
	82	12.36^a^
	123	12.16^a^
*P values*		**
LSD _0.05_		1.06
CV(%)		9.00

Where

*** = p<0.001

** = P<0.01; and Ns = P>0.05. Means followed by the same letter within the same column are not significantly different at the 5% probability level; LSD_0.05_ = least significant difference at the 5% probability level; and CV = coefficient of variation.

The highest value of pungency measured as pyruvic acid (10.71 μmol/g) was recorded for the Bombay Red variety, followed by the Russet variety (10.04 μmol/g), whereas the lowest value of pungency was recorded for the Jambar variety (8.541 μmol/g), as indicated in Table 8.

**Table 8:** Effects of variety and nitrogen on onion bulb pungency across the three locations.

Treatment	Pungency (μ mol./g)
Russet	10.04^b^
Red Coach	8.82^c^
Jambar	8.541^c^
Bombay Red	10.71^a^
Significance level	***
LSD_0.05_	0.35
**Nitrogen rate (kg ha** ^**-1**^ **)**	
0	9.45^b^
41	9.45^b^
82	9.52^ab^
123	9.71^a^
*P values*	**
LSD _0.05_	0.34
CV (%)	9.8

Where

*** = p<0.001

** = P<0.01; and Ns = P>0.05. Means followed by the same letter within the same column are not significantly different at the 5% probability level; LSD_0.05_ = least significant difference at the 5% probability level; and CV = coefficient of variation.

Among the nitrogen fertilizers, the application of 123 kg ha^-1^ nitrogen resulted in the highest values of pungency (9.71 μmol/g), which was statistically similar to the results of the application of 82 kg ha^-1^ nitrogen (9.51 μmol/g). The bulbs produced without nitrogen fertilizer presented the lowest level (9.45 μmol/g) of pungency (Table 8).

Pungency is one of the most important quality parameters of onion bulbs and is associated with the content of sulfur compounds. Pungency is influenced by onion variety, environmental conditions, and the management practices implemented [86], which is in agreement with the findings of the present study. Like in the present study, Randle [87] reported an increased level of bulb pungency with the application of nitrogen fertilizer, which could be explained by the synthesis and accumulation of sulfur-containing amino acids, which are precursors of flavoring compounds and pyruvate. According to Yoo *et al*. [88] and Gallina *et al*. [89], the pyruvic acid content is influenced by the environment, growing conditions and storage periods. Like in the present study, da Silva *et al*. [86] reported that onion pungency was affected by variety, the growing season and the nitrogen fertilizer rate.

### 3.4. Economic analysis of onion as influenced by variety and nitrogen fertilizer rate

**Fig 4:**
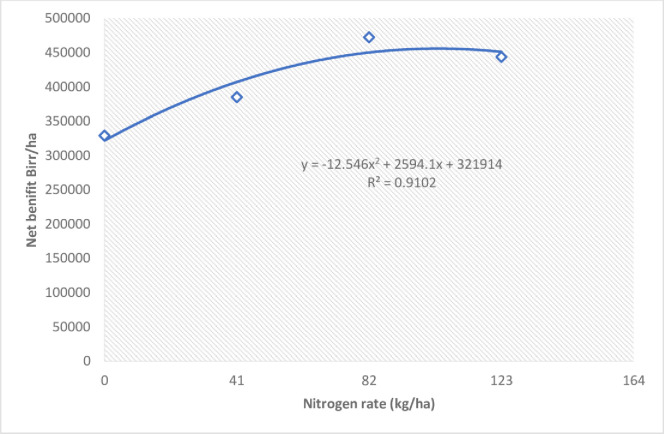
Polynomial curve fit and regression equation illustrating the relationship between nitrogen rate (kg/ha) and net benefit (Birr/ha).

The partial budget analysis indicated that the application of 82 kg ha^-1^ nitrogen fertilizer (474,745 ETB ha^-1^) and Russet (413,000 ETB ha^-1^) and Jambar (378,260 ETB ha^-1^) varieties across the three locations resulted in relatively high net benefits (Table 9). As shown in Fig 2, the application of 82 kg ha^-1^ nitrogen resulted in the highest bulb yield and the highest net return (Fig 4). An additional increase in the nitrogen rate beyond 82 kg ha^-1^ reduced the net return of onion (Fig 4), which could be considered optimal for onion production in the study area.

**Table 9:** Partial budget analysis for onion production as influenced by variety and nitrogen fertilizer level across the three locations.

Treatments	MY (t ha^-1^)	AMY (t ha^-1^)	GB (Birr/ha)	TVC (Birr/ha)	Net benefit (Birr/ha)	Dominance analysis	MRR (%)
**Variety**							
Bombay Red	19.86	17.874	357480	18000	339480		-
Jambar	24.57	22.113	442260	64000	378260		84.3
Russet	26.5	23.85	477000	64000	413000		159.83
Red Coach	21.84	19.656	393120	64000	329120	**D**	-
**Nitrogen rate (kg ha** ^**-1**^ **)**							
0	19.09	17.181	343620	0	343620	-	-
41	22.39	20.151	403020	3557.6	399462		1569.665
82	26.77	24.093	481860	7115.2	474745		2116.101
123	24.51	22.059	441180	10672.8	430507	**D**	-

Note: MY = marketable yield; AMY = adjusted marketable yield; GB = gross benefit, TVC = total variable cost, NB = net benefit; D = dominated and MRR = marginal rate of return.

## 4 Conclusions and recommendations

The onion growth and yield parameters were influenced by the nitrogen fertilizer rate and variety across the three locations. Compared with the open pollinated Bombay Red variety, the hybrid varieties (Russet and Jambar) performed well in terms of bulb diameter, bulb weight, total yield, and marketable bulb yield parameters. In general, the highest growth and yield parameters of onion were recorded when nitrogen fertilizer was supplied at a rate of 82 kg ha ^-1^. The onion plants supplied with 82 kg ha^-1^ nitrogen recorded the highest marketable bulb yield of 26.77. The experiment indicated that too much N (above 82 kg ha^-1)^ leads to decreased yield; hence, excess N is lost to the environment.

The Russet and Jambar varieties recorded the highest marketable bulb yields, with values of 26.50 t ha^-1^ and 24.57 t ha^-1^, respectively. The Russet and Jamber varieties and the 82 kg ha^-1^ nitrogen rate also recorded the greatest net benefit across all three locations. Given the significant economic benefits associated with their higher marketable yields, Russet and Jambar are recommended for production, particularly in northwest Amhara, where yield is prioritized. Therefore, Jambar and Russet varieties and the application of 82 kg ha^-1^ nitrogen fertilizer, are recommended for the economical production of onions in areas of the study area with similar agroecology. Since this study was the first of its kind in the study area, other hybrid onion varieties should be considered, and agronomic practices such as spacing and phosphorous fertilizer should be optimized in future research. In addition, further research is required to enhance the quality characteristics of the high yielding varieties.

## Supporting information

S1 Table Mean squares and significance level for onion traits tested. (DOCX)

S1 Data (XLS)
