# Supplementary material for: Enhancing bulb yield through nitrogen fertilization and the use of hybrid onion (Alluim cepa L.) varieties in northwest Ethiopia
Source: PLoS One. 2024 Oct 24;19(10):e0312394. doi: 10.1371/journal.pone.0312394 (PMC11500932; doi:10.1371/journal.pone.0312394)
Supplement: S1 Table — (DOCX) [file pone.0312394.s001.docx]

Appendix

Appendix Table 1. Mean squares and significance level for onion traits tested

| Traits | Block(L) | Variety (Var) | Nitrogen(N) | Var*N |
| --- | --- | --- | --- | --- |
| df | 6 | 3 | 3 | 9 |
| DPM | 53.63ns | 1143.00*** | 125.5** | 7.58ns |
| PH | 95.67** | 51.433* | 186.387*** | 6.382ns |
| LNPP | 1.54ns | 11.75*** | 5.20** | 0.12ns |
| BD | 20.88ns | 78.25** | 1084.97*** | 75.99ns |
| AVBW | 428.92** | 1178.36*** | 3080.09*** | 58.89ns |
| TYTH | 21.12ns | 297.16*** | 373.20*** | 4.32ns |
| UMYP | 0.03ns | 0.15*** | 0.096*** | 0.013ns |
| MYTH | 6.75** | 5.33** | 0.64* | 1.08ns |
| TSS | 0.02ns | 116.46*** | 8.17*** | 2.49** |
| Pungency | 0.73ns | 38.28*** | 0.72** | 0.006ns |

L=location, df=degrees of freedom, DPM = days to physiological maturity, PH = plant height, LNPP = leaf number per plant, BD = bulb dimeter, AVBW = average bulb weight, TYTH = total yield ton per hectare, UMYP = unmarketable yield percentage, MYTH = marketable yield ton per hectare and TSS = total soluble solids; ****= p<0.001; ** = P<0.01; and Ns = P>0.05.* Means followed by the same letter within the same column are not significantly different at 5% probability level
